# A comparison of thought and perception disorders in borderline personality disorder and schizophrenia: psychotic experiences as a reaction to impaired social functioning

**DOI:** 10.1186/s12888-014-0239-2

**Published:** 2014-10-03

**Authors:** Francesco Oliva, Marinella Dalmotto, Elvezio Pirfo, Pier Maria Furlan, Rocco Luigi Picci

**Affiliations:** Department of Mental Heath “G. Maccacaro”, Azienda Sanitaria Locale TO2, Turin, Italy; Department of Clinical and Biological Sciences, “San Luigi Gonzaga” Medical School, University of Turin, Regione Gonzole 10, 10043 Orbassano TO Turin, Italy

**Keywords:** Borderline personality disorder, Schizophrenia, Thought disorder, Perception disorder, Psychotic-like symptoms, Quasi-psychotic symptoms, Social functioning, Non-delusional paranoia, DSM

## Abstract

**Background:**

Although previous studies suggest a high frequency of psychotic symptoms in DSM-IV Borderline Personality Disorder (BPD) there is currently no consensus on their prevalence and characteristics (type, frequency, duration, location etc.). Similarly, there are few papers addressing psychotic reactivity, the crucial aspect of BPD included in the ninth criterion for DSM-IV BPD, which remained unchanged in DSM-IV-TR and DSM-5. The purposes of the present study were to compare thought and perception disorders in patients with DSM-IV BPD and schizophrenia (SC), investigating their relationship with social functioning.

**Methods:**

Thought and perception disorders and social functioning over the previous two years were assessed by the Diagnostic Interview for Borderline Revised (DIB-R) and Personal and Social Performance scale (PSP) respectively in outpatients with DSM-IV BPD (n = 28) or DSM-IV SC (n = 28).

**Results:**

Quasi-psychotic thought (i.e. transient, circumscribed and atypical psychotic experiences) was more frequent in BPD (BPD = 82.1%, SC = 50%, *p* = 0.024); whereas true psychotic thought (i.e. Schneiderian first-rank, prolonged, widespread and bizarre psychotic symptoms) was more frequent in SC (SC = 100%, BPD = 46.4%, *p* < 0.001). However both types of psychotic features were prevalent in both groups. Non-delusional paranoia (e.g. undue suspiciousness and ideas of references) was ubiquitous but was more severe in BPD than SC patients (U(54) = 203.5, *p* = 0.001). In the BPD group there was a strong negative correlation between personal and social functioning and non-delusional paranoia (τ(28) = 0.544, *p* = 0.002) and level of personal and social functioning was a significant predictor of the severity of non-delusional paranoia only in the BPD group (β = −0.16, t(23) = 2.90, *p* = 0.008).

**Conclusions:**

BPD patients reported less severe psychotic experiences with more frequent quasi-psychotic thought, less frequent true psychotic thought and more severe non-delusional paranoia than SC patients. Interpersonal functioning seems to predict non-delusional paranoia in BPD, which would validate the “stress-related paranoid ideation”, included in the ninth diagnostic criterion for DSM-IV and DSM-5 BPD. PBD patients had higher scores on the psychotic experiences subscale that support the use of a dimensional assessment of the severity of thought and perception disorders, for example the Clinician-Rated Dimensions of Psychosis Symptom Severity introduced in DSM-5, Section III.

## Background

According to the first Stern’s definition [1], Borderline Personality Disorder (BPD) is a nosographic entity on the border between neurosis and psychosis. This definition received support from several authors who worked on pseudo-neurotic disorders which were too severe to benefit from classical psychoanalytic treatment, or psychotic-like disorders which were insufficiently severe to be considered psychotic disorders [2-5]. According to psychodynamic perspectives, patients with BPD are usually capable of reality testing, but may lose this capacity and suffer transient psychotic episodes under severe stress (especially when they are exposed to real or imagined abandonment [6]), during psychotherapy (so called ‘transference psychosis’) [5,7,8], or under the influence of alcohol or other drugs [7,9-12].

This was consistent with both DSM-III (Diagnostic and Statistical Manual of Mental Disorders, 3rd ed.) [13] and DSM-III-R [14], which reported that in BPD transient psychotic symptoms may occur during periods of extreme stress; however, none of the eight criteria for DSM-III/ DSM-III-R BPD referred to psychotic features and instead the classification of concurrent psychotic symptoms was covered by Axis I psychotic disorders. In contrast with DSM-III and DSM-III-R, some authors reported high rates of some peculiar psychotic-like features when assessing DSM-III and DSM-III-R BPD [15–20]. Zanarini and colleagues [21] conducted an in-depth evaluation of the cognitive features of BPD comparing three clinical groups (BPD, n = 50; other personality disorders, n = 55; schizophrenia, n = 32) with healthy controls (n = 46). They used the Diagnostic Interview for Borderline Revised (DIB-R) [22] to assess psychopathology over the previous two years and the Structured Clinical Interview for DSM-III (SCID) [23] to evaluate lifetime psychotic episodes. Using the DIB-R, quasi-psychotic thought characterized by transient (lasting less than two days), circumscribed (affecting not more than two areas of life ) or atypical psychotic symptoms (based on reality or totally fantastic), were observed in 20 patients (40%) with BPD, one patient (1.8%) with other personality disorder (PD), and none of the patients with schizophrenia (SC), whereas only patients with SC reported true psychotic thought, defined as prolonged, widespread, bizarre and stereotypical psychotic symptoms, i.e. Schneiderian first-rank symptoms. Using SCID all 32 patients with SC, seven patients (14%) with BPD and two patients (2.6%) with other PD reported one or more lifetime psychotic episodes. In BPD patients these occurred only in the context of either co-morbid substance use disorder or co-morbid affective disorder as previously reported by Pope and colleagues [17]. These findings [21] confirmed the predicted specificity of true psychotic thought for SC and extended previous reports of the prevalence of transient psychotic-like symptoms in BPD, suggesting that quasi-psychotic thought was a highly discriminant sign and therefore “virtually pathognonomic” for BPD.

In DSM-IV [24] the section on BPD was supplemented: a ninth criterion for a categorical diagnosis of BPD, the presence of “transient stress-related paranoid ideation or severe dissociative symptoms” was added; it was stated that the severity and duration of psychotic symptoms should not be such as to warrant an additional diagnosis of psychotic disorder, and the development of psychotic-like symptoms (e.g. hallucinations, body-image distortion, ideas of references, and hypnagogic phenomena) during times of stress was recognized as an associated feature supporting diagnosis of BPD. However no explicit reference to “regression proneness” or the loss of reality testing was included in DSM-IV and this drew a certain amount of criticism [11].

Since the introduction of DSM-IV, some studies have reported high rates of psychotic symptoms in BPD [25–30], but few of these studies used a structured interview capable of discriminating between quasi-psychotic thoughts and true psychotic thoughts in the manner of DIB-R. For instance, Yee and colleagues [29] found that 29.2% of 171 patients being treated for DSM-IV BPD reported ‘hearing voices’ on Symptom-Checklist-90 (SCL-90) [31], but an in-depth evaluation (covering the frequency, duration and type of perception disorder) was only conducted in a non-random subsample of ten cases. On this basis the authors suggested that hallucinatory phenomena were an ongoing, pervasive feature of BPD and were not atypical for this patient group.

Two other studies [27,28] compared patients with BPD and SC using SCID-II to diagnose DSM-IV BPD and the Psychotic Symptom Rating Scale (PSYRATS) [32] to assess the subjective characteristics of hallucinations and delusions (e.g. frequency, duration, amount of distress etc.). Kingdon and colleagues [27] found that 29% of 33 patients with BPD experienced paranoid delusions compared with 61% of 59 patients with SC and 65% of 19 patients with a diagnosis of both BPD and SC. Only 50% of patients with BPD experienced auditory hallucinations compared with 66% of SC patients and 90% of patients with SC and BPD; there were no significant differences in the frequency, duration or location of hallucinations; however substance use was not taken into account in this study. Slotema and colleagues [28] explicitly excluded patients with alcohol abuse, substance use or current delusions; they recruited a sample of 38 women with BPD, 51 women with SC and 66 healthy women and confirmed that the frequency, duration, and location of auditory verbal hallucinations were similar in patients with BPD and SC.

More recently, Pearse and colleagues [30] found highest lifetime prevalence of psychotic symptoms in DSM-IV BPD (80%), using the Present State Examination (PSE) [33], though patients with affective and substance use disorders were not excluded and psychotic symptoms met DSM-IV SC criteria in 10% of the sample.

In summary, although previous studies suggest so much high frequency of psychotic symptoms in DSM-IV BPD than it has been recently proposed to avoid terms like pseudo-psychotic or quasi-psychotic [34], there is currently no consensus on their prevalence and characteristics (type, frequency, duration, location etc.). Since the introduction of the ninth criterion by the DSM-IV task force - which remained unchanged in DSM-IV-TR [35] and DSM-5 [36] - no study has explicitly estimated the prevalence of quasi-psychotic thought in BPD.

Similarly, there are few papers addressing psychotic reactivity, the crucial aspect of BPD included in the ninth criterion for DSM-IV BPD. Suzuki and colleagues [37] conducted an in-depth evaluation of five patients with BPD and psychotic symptoms that provided support for the psychodynamic perspective [3,5,7], which assigns interpersonal functioning the crucial role in the development of both delusion and hallucinations. These authors also distinguished between different types of symptoms: they suggested that paranoid delusions were due to the projection of rage and hostile feelings onto other people, whereas hallucinations tended to occur as a result of avoidance of an interpersonal relationship, and the presence of both delusions and hallucinations was a consequence of interpersonal problems in patients with a passive attitude.

More recently, Glaser and colleagues [38] explicitly investigated stress-related psychotic responses in a sample of 65 patients with BPD and, although they did not explicitly refer to the psychodynamic framework, they reported that enhanced psychotic reactivity to the stresses of daily life was more common in BPD than in psychotic disorders or Cluster C personality disorders. They also found a wide range of stress-related symptoms, which they termed reality disturbances, that seem to fit better with the broader definition included in the DSM-IV-TR description of BPD (psychotic-like symptoms such as hallucinations, body-image distortion, ideas of references, hypnagogic phenomena) than with the narrowly defined ninth diagnostic criterion for DSM-IV-TR BPD (i.e. paranoid and dissociative ideation).

In order to assess whether the transition from DSM-III to DSM-IV led to changes in reports of thought and perception disorders in BPD and whether this psychopathology is a reaction to interpersonal functioning, the aims of the present study were (1) to describe perception and thought disorders in a group of outpatients with DSM-IV BPD, comparing their symptoms with those of outpatients with DSM-IV SC; and (2) to explore relationship between these symptoms and function searching for possible correlations between perception and thought disorders and personal and social functioning in both groups and subsequently estimating the extent to which level of personal and social functioning predicts perception and thought disorders in both groups.

## Methods

### Sample and procedures

The research protocol of the present cross-sectional comparative study was approved by the Research Ethics Committee of the *Azienda Sanitaria Locale* [local health authority] *TO2* (Turin, Italy) and therefore the study was conducted in accordance with the Helsinki Declaration.

All outpatients who accessed the *Centro di Salute Mentale – Distretto I* (CSM; District 1 Centre for Mental Health) of the *Giulio Maccacaro* Mental Health Department, Turin from January 2011 to March 2011 were invited to participate in the study, but only those who met the eligibility criteria and gave written informed consent were enrolled. Eligibility criteria were as follows: (1) under he care of CSM for at least two years for a BPD or SC, (2) aged 18–45 years, and (3) completed primary education.

All enrolled patients were screened using the Structured Clinical Interview for DSM-IV Axis I (SCID-I) [39] and the Structured Clinical Interview for DSM-IV Axis II (SCID-II) [40] and assigned to the BPD group or SC group; patients with both SC and BPD were excluded from the study as were patients with current DSM-IV substance use disorder or current DSM-IV mood disorder. Although all patients accessing the CSM were treated in accordance with evidence-based recommendations provided by American Psychiatric Association Practice Guidelines [41], information about pharmacological treatment were limited to current antipsychotic treatment only, and they were collected from clinical records as dichotomous variable (yes/no).

### Assessment tools

SCID-I and SCID-II are semi-structured interview protocols with a modular structure based on DSM-IV Axis I and DSM-IV Axis II respectively. In this study they were used to diagnose BPD, SC and substance use disorders according to DSM-IV.

Psychotic symptoms were assessed using the cognitive section of the DIB-R [22]. This is a one hundred and nineteen-item, semi-structured interview that evaluates four main areas of BPD phenomenology (affective, cognitive, impulsive and interpersonal), over the previous two years, it is currently considered the international scientific standard for the assessment of BPD [42].

The cognitive section consists of 27 items (each item assess a single type of cognition) grouped into three subsections: odd thinking/ unusual perceptual experiences (9 items), non-delusional paranoia (3 items) and psychotic experiences (15 items). The first two subsections assess disturbed but non-psychotic thought and the psychotic experiences subsection assesses quasi-psychotic thought and true psychotic thought.

In the present study, the assessment of psychotic features considered the total score for the DIB-R cognitive section, the prevalence of each type of thought and scores on the DIB-R cognitive subsections.

Personal and social functioning were assessed with the Personal and Social Performance scale (PSP) [43]. This is a brief structured clinical interview protocol that evaluates four main areas: socially useful activities (area A), personal and social relationships (area B), self-care (area C), and disturbing and aggressive behaviors (area D). The PSP provides a score between 1 and 100 using a 6-point severity scale for each area; higher scores represent better personal and social functioning. The validity and reliability of the PSP have already been evaluated [44,45]. Although the standard PSP investigates functioning over the past month, the PSP manual [43] states that longer time intervals can be specified; we used a time interval of two years.

### Statistical analysis

All analyses were conducted using IBM SPSS Statistics for MACOS (Version 19, Armonk: IBM Corporation).

Pearson’s χ^2^ test was used for group comparison of categorical variables. Fisher’s exact test replaced the χ^2^ test when any expected frequency was less than or equal to five. The normality of the distribution of continuous variables (i.e. age, DIBR scores, and PSP scores) was evaluated by Shapiro-Wilk’s test. Normally distributed variables were compared using an independent samples t-test and non-normally distributed variables were evaluated using the non-parametric Mann–Whitney U test.

The association between thought and perception disorders and functioning was examined by constructing separate correlation matrices for the BPD group and SC group, using the DIB-R cognitive section scores, the DIB-R cognitive subsections scores (odd thinking/unusual perceptual experiences, non-delusional paranoia, psychotic experiences), the PSP total scores, and the four PSP area scores (socially useful activities, personal and social relationships, self-care, disturbing and aggressive behaviors). The strength of bivariate correlations was reported as Pearson’s r coefficient or as Kendall’s tau (*τ*) coefficient, depending on whether distribution of the variables was normal or non-normal.

Linear regression analysis consisted of three distinct models for each group (BPD and SC); each model used a different DIB-R subsection score as the dependent variable. All models were adjusted for significant group differences on the socio-demographic variables and included the four PSP domains as independent variables. A *p*-value of 0.05 was used to designate statistical significance.

## Results

A total of 64 patients gave their informed consent and fulfilled the eligibility criteria; eight (12.5%) patients were excluded because they had a current DSM-IV substance use disorder (n = 4, 6.2%) or a current DSM-IV mood disorder (n = 2, 3.1%) or a diagnosis of both BPD and SC (n = 2, 3.1%).

Following screening with SCID-I and SCID-II 28 (50%) patients were assigned to the BPD group and 28 patients (50%) were assigned to the SC group. All patients in the SC group were taking antipsychotic drugs in accordance with evidence-based recommendations provided by American Psychiatric Association Practice Guidelines [41], but none of the BPD patients were taking antipsychotic drugs.

There were no significant group differences in gender, marital status, education or employment (Table 1). However, the mean age of the SC group was significantly higher than that of the BPD group.

**Table 1:** **Socio-demographic characteristics of the BPD group and SC group**

	**SC n(%)**	**BPD n(%)**	***p***
Gender			0.788^a^
Female	15(53.6)	16(57.1)	
Male	13(46.4)	12(42.9)	
Marital status			0.513^b^
Single (never married)	21(75.0)	18(64.3)	
Married	2(7.1)	4(14.3)	
Separated/Divorced	4(14.3)	6(21.4)	
Widowed	1(3.6)	0(0.0)	
Education, years			0.119^b^
1–5	0(0.0)	3(10.7)	
6–8	13(46.4)	17(60.7)	
9–13	12(42.9)	7(25.0)	
>13	3(10.7)	1(3.6)	
Employment			1^a^
Employed	8(28.6)	8(28.6)	
Unemployed	20(71.4)	20(71.4)	
	Mean(SD)	Mean(SD)	
Age, years	40.1(5.8)	35.2(8.08)	0.012^c^

SC: schizophrenia; BPD: Borderline Personality Disorder.

^a^Pearson’s χ^2^ test.

^b^Fisher’s exact test.

^c^Pearson’s independent samples t-test.

### Thought and perception disorders

The DIB-R results are presented in Table 2. The mean score for the cognitive section and the psychotic experiences subsection were significantly lower for the BPD group than the SC group (DIB-R cognitive section: t (54) = 2.79, *p* = 0.007; psychotic experiences subsection: U (54) = 75.50, *p* < 0.001). The BPD group also had a significantly higher mean score on the non-delusional paranoia subsection (U (54) = 203.50, *p* = 0.001). All the patients in the BPD group reported at least one symptom of non-delusional paranoia compared to 25 patients (89.3%) in the SC group (Fisher’s exact test, *p* = 0.236). There were no significant group differences in the mean scores on the odd thinking/unusual perceptual experiences subsection (t (54) = −0.20, *p* = 0.842) and in both groups the same proportion of patients (n = 24, 85.7%) reported at least one type of odd thinking/unusual perceptual experience.

**Table 2:** **DIB-R cognitive section and DIB-R cognitive subsections scores for the BPD group and SC group**

	**SC (n = 28)**	**BPD (n = 28)**	
**DIB-R scores**	**Mean(SD)**	**Mean(SD)**	***p***
Cognitive section	26.29(9.20)	20.11(7.22)	0.007^a^
Odd thinking/unusual perceptual experiences	6.04(3.96)	6.25(4.06)	0.842^a^
Non-delusional paranoia	4.00(1.36)	5.11(0.92)	0.001^b^
Psychotic experiences	12.04(4.73)	4.68(3.41)	<0.001^b^

SC: schizophrenia; BPD: Borderline Personality Disorder.

^a^Pearson’s independent samples t-test.

^b^Mann–Whitney U test.

Results for the prevalence of different types of disordered thought are presented in Table 3. All the SC patients but only slightly less than half of the BPD patients had experienced true psychotic thought during the previous two years (Fisher’s exact test, *p* < 0.001). Conversely quasi-psychotic thought was significantly more frequent in patients with BPD than with SC (χ^2^ (1) = 5.10, *p* = 0.024). All the patients had experienced disturbed but non-psychotic thought, but only four (14.3%) with BPD and none with SC had experienced only this type of disordered thought during the previous two years.

**Table 3:** **Prevalence of DIB-R types of thought in the BPD group and SC group**

	**SC (n = 28)**	**BPD (n = 28)**	
**DIB-R types of thought**	**n(%)**	**n(%)**	***p***
True psychotic thought	28(100)	13(46.4)	<0.001^a^
Quasi-psychotic thought	14(50)	23(82.1)	0.024^b^
Disturbed but non-psychotic thought	28(100)	28(100)	1

SC = Schizophrenia; BPD = Borderline Personality Disorder.

^a^Fisher’s exact test.

^b^Pearson’s χ^2^ test.

### Personal and social functioning

The personal and social functioning (Table 4) of patients with BPD did not differ from that of SC patients in terms of PSP total score (U (54) = 307.00, *p* = 0.168), socially useful activities score (PSP area A, U (54) = 375.00, *p* = 0.860), personal and social relationships score (PSP area B, U (54) = 392.00, *p* = 1) or self-care score (PSP area C, U (54) = 387.50, *p* = 0.850). Only the disturbing and aggressive behaviors score (PSP area D) was significantly worse in the BPD group (U (54) = 178.50, *p* < 0.001).

**Table 4:** **PSP scores for the BPD group and SC group**

	**SC (n = 28)**		**DBP (n = 28)**		
**PSP area**	**Media(SD)**	**Median(IQR)**	**Media(SD)**	**Median(IQR)**	***p***
Area A	3.32(0.77)	3(1)	3.35(0.73)	3.5(1)	0.860^a^
Area B	3.39(0.57)	3(1)	3.39(0.57)	3(1)	1^a^
Area C	2.18(0.67)	2(1)	2.21(0.74)	2(2)	0.850^a^
Area D	2.07(0.94)	2(1)	3(0.72)	3(2)	<0.001^a^
PSP total score	52.68(9.28)	50(14)	48.93(10.75)	47.5(20)	0.168^a^

SC: schizophrenia; BPD: Borderline Personality Disorder; IQR: interquartile range; Area A: useful activities; Area B: personal and social relationships; Area C: self-care; Area D: disturbing and aggressive behaviors.

^a^Mann–Whitney U test.

### Relationship between thought and perception disorders and functioning areas

In the BPD group (Table 5), no significant bivariate correlations were found between the three DIB-R subsections, whereas in the SC group (Table 6) the non-delusional paranoia subsection score was significantly correlated with psychotic experiences subsection score (*τ* (54) = 0.45, *p* = 0.001).

**Table 5:** **Correlations between DIB-R and PSP in the BPD group**

	**DIB-R C**	**PODD**	**PPAR**	**PPSY**	**PSP tot**	**Area A**	**Area B**	**Area C**	**Area D**
DIB-R C	1	-	-	-	-	-	-	-	-
PODD	0.839^**^	1	-	-	-	-	-	-	-
PPAR	0.401^**^	0.179	1	-	-	-	-	-	-
PPSY	0.518^**^	0.216	0.180	1	-	-	-	-	-
PSP tot	−0.198	−0.102	−0.301	−0.114	1	-	-	-	-
Area A	0.099	0.062	0.221	0.039	−0.546^**^	1	-	-	-
Area B	0.168	0.066	0.544^**^	0.052	−0.456^**^	0.430^*^	1	-	-
Area C	−0.061	−0.123	0.339^*^	−0.003	−0.496^**^	0.438^*^	0.306	1	-
Area D	0.231	0.212	0.067	0.048	−0.497^**^	0.262	0.089	0.141	1

DIB-R C: DIB-R cognitive section; PODD: odd thinking/unusual perceptual experiences; PPAR: non-delusional paranoia; PPSI: psychotic experiences; PSP tot: PSP total score; Area A: useful activities; Area B: personal and social relationships; Area C: self-care; Area D: disturbing and aggressive behaviors.

**Kendall’s tau-b, *p* < 0.01 (2-tailed).

*Kendall’s tau-b, *p* < 0.05 (2-tailed).

**Table 6:** **Correlations between DIB-R and PSP in the SC group**

	**DIB-R C**	**PODD**	**PPAR**	**PPSY**	**PSP tot**	**Area A**	**Area B**	**Area C**	**Area D**
DIB-R C	1	-	-	-	-	-	-	-	-
PODD	0.885^**^	1	-	-	-	-	-	-	-
PPAR	0.349^*^	0.274	1	-	-	-	-	-	-
PPSY	0.695^**^	0.447^**^	0.158	1	-	-	-	-	-
PSP tot	−0.076	−0.062	−0.016	−0.130	1	-	-	-	-
Area A	−0.099	−0.141	−0.056	0.047	−0.748^**^	1	-	-	-
Area B	0.196	0.092	0.122	0.242	−0.575^**^	0.390^*^	1	-	-
Area C	0.017	0.028	−0.039	0.087	−0.508^**^	0.403^*^	0.247	1	-
Area D	−0.078	0.045	−0.061	−0.111	−0.537^**^	0.433^*^	0.259	0.611^**^	1

DIB-R C: DIB-R cognitive section; PODD: odd thinking/unusual perceptual experiences; PPAR: non-delusional paranoia; PPSI: psychotic experiences; PSP tot: PSP total score; Area A: useful activities; Area B: personal and social relationships; Area C: self-care; Area D: disturbing and aggressive behaviors.

**Kendall’s tau-b, *p* < 0.01 (2-tailed).

*Kendall’s tau-b, *p* < 0.05 (2-tailed).

In the BPD group (Table 5) the PSP area A score (socially useful activities) was significantly correlated with the PSP area B score (personal and social relationships) and the PSP area C score (self-care), whereas in the SC group (Table 6) the PSP area A score was significantly correlated with scores for all the other areas and the PSP area C score was correlated with the PSP area D (disturbing and aggressive behaviors) score (*τ* (54) = 0.61, *p* < 0.001).

Neither the DIB-R cognitive section nor any of the DIB-R cognitive subsections were significantly correlated with PSP total score in either group. For the SC group there were no correlations between DIB-R cognitive subsections and PSP areas (Table 6); however the DIB-R non-delusional paranoia subsection was significantly correlated with PSP area B (*τ* (54) = 0.54, *p* = 0.002) and, to a lesser extent with PSP area C (*τ* (54) = 0.34, *p* = 0.047).

### Predicting value of functioning areas for thought and perception disorders

The linear regression analysis showed that for the SC group the proportion of the variance explained by all the models was very low (odd thinking/unusual perceptual experiences: R^2^ = 0.09, F (4,23) = 0.68, *p* = 0.680; non-delusional paranoia: R^2^ = 0.05, F (4,23) = 0.35, *p* = 0.840; psychotic experiences: R^2^ = 0.20, F (4,23) = 1.44, *p* = 0.253). Furthermore none of four PSP areas was a significant predictor of scores on any of the DIB-R subsections.

For the BPD group two models explained low proportions of the variance in the dependent variable and did not reach statistical significance (odd thinking/ unusual perceptual experiences: R^2^ = 0.14, F (4,23) = 0.91, *p* = 0.473; psychotic experiences: R^2^ = 0.10, F (4,23) = 0.65, *p* = 0.631) and none of PSP areas showed significant predictive value. However, as shown in Table 7, the model with non-delusional paranoia as the dependent variable explained 36.1% of the variance (R^2^ = 0.36, F = 3.25, *p* = 0.030) and in this model PSP area B score significantly predicted non-delusional paranoia (β = −0.16, t (23) = 2.90, *p* = 0.008).

**Table 7:** **Linear regression of PSP areas on non-delusional paranoia in the BPD group (n = 28)** ^**a**^

**R** ^**2**^ **= 0.361, Adjusted R** ^**2**^ **= 0.250, F(4,23) = 3.254,** ***p*** = 0.030*			
**Independent variables**	**β**	**t**	***p***
Area A	0.076	−0.789	0.438
Area B	−0.165	2.903	0.008*
Area C	0.549	1.249	0.224
Area D	0.238	0.435	0.667

Area A: useful activities; Area B: personal and social relationships; Area C: self-care; Area D: disturbing and aggressive behaviors.

^a^adjusted for socio-demographic variables that differed significantly between groups.

*p < 0.05 (2-tailed).

## Discussion

As expected, patients with BPD showed less severe symptoms than those with SC, as indicated by higher scores on both DIB-R cognitive section and DIB-R psychotic experience subsection.

The overall cognitive pattern shown by BPD patients in our investigation seems to be roughly consistent with that reported by previous authors who used the same tool to assess DSM-III BPD [17,20,21]: the BPD group presented (a) ubiquitous odd thinking, unusual perceptual experiences and non-delusional paranoia; (b) frequent transient, circumscribed and atypical psychotic experiences (i.e. quasi-psychotic thought), and (c) some ongoing, bizarre, Schneiderian first-rank symptoms (i.e. true psychotic thought).

Zanarini and colleagues [21] suggested that quasi-psychotic thought was “virtually pathognonomic” for BPD because of its high prevalence in their BPD group (40%) and its absence from their SC group (0%) whereas in the present study this type of thought, although more frequent in the BPD group (82.1%), was also encountered in around half the SC group and therefore does not seem to discriminate between BPD and SC. The high prevalence of quasi-psychotic thought in our SC group compared to that of Zanarini and colleagues [21] might be explained by the difference in setting (outpatients only vs. outpatients and community inpatients) or by differences in psychopharmacological treatments (e.g. first- vs. second- generation antipsychotics, oral vs. long- acting injectable agents etc.), specifically antipsychotic agents might have controlled and altered the clinical expression of some psychotic episodes in our SC patients.

The prevalence of true psychotic thought in DSM-III BPD reported in previous studies which used DIB-R ranged from 0% [21] to 3.8% [46], whereas in the present study almost half the DSM-IV BPD group (46.4%) reported at least one true psychotic experience during the previous two years. This finding seems to be consistent with some recent studies of psychopathology in DSM-IV BPD, which reported a noteworthy prevalence of persistent psychotic symptoms, even if a careful comparison is complicated by differences in samples and methods. For instance, Kingdom and colleagues [27] reported that 29% of BPD patients had paranoid delusions according to SCID-I and 50% had auditory hallucinations according to PSYRATS, but this study did not exclude or take into account substance use disorder; they failed to find significant differences in the persistence of hallucinations in BPD and SC. Yee and colleagues [29] reported that 29.2% of 171 patients with BPD assessed by SCL-90 heard voices, but their qualitative assessment of perception disorders was carried out son a non-random subsample of ten cases. Pearse and colleagues [30] found highest lifetime prevalence of psychotic symptoms in DSM-IV BPD (80%), but 75% of the sample had a history of alcohol and/or illicit drug use, and affective disorders were not excluded.

The prevalence of quasi-psychotic thought in our sample of DSM-IV BPD was also higher than that found by Zanarini and colleagues [21] in a DSM-III BPD sample using the same assessment tool (82.1% vs. 40%).

Taken together, these findings suggest that changes in the DSM definition of BPD (i.e. the introduction of the ninth diagnostic criterion and the more complete description of BPD) may have increased its sensitivity to psychotic and psychotic-like symptoms leading to the inclusion of patients who have had psychotic episodes that were not severe enough to meet criteria for either brief psychotic disorder or another Axis I psychotic disorder (e.g. the criteria proposed by DSM-5 for Attenuated Psychosis Syndrome). Given that in the present study the mean psychotic experiences score for the BPD group was one third of the score for the SC group, we suggest that a severity scale for psychotic symptoms, such as the Clinician-Rated Dimensions of Psychosis Symptom Severity introduced in DSM-5, might be used to improve the assessment of psychotic symptoms in BPD as it would allow a more careful distinction between the psychotic features of BPD and SC. However, placing this dimensional scale in DSM-5 Section III is unlikely to be the most effective way to encourage its use, as Maj [47] has already noted.

Overall functioning was similar in the two groups; however aggressive acts and socially disturbing behavior were more frequent in the BPD group than the SC group, even considering that antipsychotic therapy may have had a crucial role in decreasing violent behavior in SC patients [48].

Our most important finding about the relationship between thought disorders and functioning concerns non-delusional paranoia. Not only were symptoms of non-delusional paranoia more severe and more frequent in the BPD group than SC group, consistent with the results of Zanarini and colleagues [21], but their severity was correlated with and predicted by the level of personal and social functioning in the BPD group, but not the SC group. These results are consistent with the most influential psychodynamic perspectives [7,9,10,12] in suggesting that severe difficulties in interpersonal functioning induce the development of undue suspiciousness, ideas of references and other symptoms of non-delusional paranoia in BPD patients.

Despite the differences in methods, our results appear to be consistent with those of Suzuki and colleagues [37] and Glaser and colleagues [38] and support the concept of stress-related thought disorder. However in the present study there was neither a correlation between non-delusional paranoia and psychotic experiences nor any functioning areas worked as predictor for psychotic experience, so low interpersonal functioning did not appear to induce psychotic experiences in BPD patients and thus, unlike Glaser and colleagues [38], we found no evidence in our BPD group of either true psychotic reactivity or quasi-psychotic reactivity, only stress-related non-delusional paranoia.

Our findings are therefore consistent with the reference to “stress-related paranoid ideation” included in the ninth diagnostic criterion for DSM-IV BPD and maintained in DSM-5, and suggest that it is still a valid and essential discriminating feature for BPD.

The lack of a prospective longitudinal assessment of functioning and psychopathology is an important limitation of the present study; this made it impossible to infer a true causal relationship between the interpersonal functioning and non-delusional paranoia; we can only suggest that level of interpersonal functioning is associated with the severity of non-delusional paranoia over the last two years, we could not determine whether the development of non-delusional paranoia in BPD patients was due to a worsening of personal or social functioning.

In view of the strong association between psychotic disorders and substances use disorder [17,21], the administration of a urine drug test alongside SCID-I would have enhanced the accuracy of sample selection and thus the characterization of thought and perception disorders in the two diagnostic groups.

Furthermore, the limited sample size and the lack of information about non-antipsychotic pharmacological treatment assumed by patients should be considered as two other limitations of the present study since they both could affect the accuracy of results.

Future research on BPD might investigate the relationship between personal and social functioning, non-delusional paranoia, and thought and perception disorders prospectively in order to determine whether non-delusional paranoia is due to changes in interpersonal functioning and whether and how it may evolve into quasi- or true psychotic experience.

## Conclusion

BPD patients reported less severe psychotic experiences with less frequent true psychotic thought, more frequent quasi-psychotic thought, and more severe non-delusional paranoia than SC patients. Interpersonal functioning seems to predict non-delusional paranoia in BPD, which would validate the “stress-related paranoid ideation”, included in the ninth diagnostic criterion for DSM-IV and DSM-5 BPD. PBD patients had higher scores on the psychotic experiences subscale that support the use of a dimensional assessment of the severity of thought and perception disorders, for example the Clinician-Rated Dimensions of Psychosis Symptom Severity introduced in DSM-5, Section III.

These results are consistent with the most influential psychodynamic perspectives in suggesting that severe difficulties in interpersonal functioning induce the development of undue suspiciousness, ideas of references and other symptoms of non-delusional paranoia in BPD patients.
